# Conflict Nephrology: War and Natural Disasters

**DOI:** 10.34067/KID.0000000000000071

**Published:** 2023-02-10

**Authors:** Tulasi Gopolan, Claudia Michelle Ornelas-Brauer, Tarek Barbar, Zain Mithani, Jeffrey Silberzweig

**Affiliations:** 1 University of Texas Southwestern School of Medicine, Dallas, Texas; 2 Division of Nephrology and Hypertension, Department of Medicine, Weill Cornell Medicine, New York, New York; 3 Idaho Kidney Institute, Chubbuck, Idaho; 4 Division of Nephrology, Department of Medicine, Leonard Miller School of Medicine at the University of Miami, Miami, Florida; 5 The Rogosin Institute, New York, New York

**Keywords:** dialysis, COVID-19, emergency, natural disaster, war

## Abstract

Access to care for patients with ESKD is frequently disrupted after natural disasters, public health crises, and human conflict. Emergency preparation can mitigate the risk of harm and improve outcomes. Before Hurricane Katrina in 2005, the United States was unprepared to assist patients facing disaster. We evaluate responses to Hurricane Katrina which caused unprecedented damage to health and property in the Gulf Coast. As a result of the multitude of identified problems with the national, local, and kidney-specific responses to Katrina, new systems were created that mitigated loss after Hurricane Sandy in 2012. The improved disaster response system was no match for the coronavirus disease 2019 pandemic; real-time changes worsened the effect on highly vulnerable populations, including patients with ESKD. Similarly, preparation can only mitigate the difficulties faced by patients with ESKD living in a war zone. Government agencies need to provide tools and dialysis centers need to educate patients. Beginning with steps implemented in the aftermath of Hurricane Katrina and augmented after Hurricane Sandy, every patient with ESKD and those who care for them must begin emergency preparations before the need arises. Recognizing that it is not possible to prepare for every possible emergency, our health care systems must be ready to adapt to our ever-changing world. After reviewing the responses to previous events, we suggest steps that should be considered to improve preparations for our uncertain future.

## Introduction

Natural disasters pose challenges to emergency management systems and the health of affected populations. Patients with ESKD are especially vulnerable because they are largely an older population with multiple comorbid conditions, compromised immunity, and dependence on maintenance dialysis or immunosuppressive medications to maintain allograft function. The tenuous health of kidney patients was evident in the aftermath of the perfect storm, Hurricane Katrina, when thousands of patients missed dialysis treatments and suffered poor health outcomes.^1,2^ The lack of preparedness on the part of the government, dialysis providers, and patients led to unnecessary suffering and uncovered the need for greater investment in emergency planning, including expansion of the health care safety net and development of guidelines for disaster planning. In its wake, interconnected kidney community networks, such as the Kidney Community Emergency Response Coalition,^3^ arose as a bolster; no subsequent disaster has wreaked as much havoc.

The coronavirus disease 2019 (COVID-19) pandemic proved to be a major threat to public health worldwide. COVID-19 has accounted for more than 92 million cases and 1,000,000 deaths in the United States.^4^ The approximately 500,000 Americans with ESKD were among the most vulnerable because most congregate to receive in-center hemodialysis treatments thrice weekly. As was the case before Hurricane Katrina, the United States was not adequately prepared.

Man-made disasters, such as those brought on by the hostilities in Ukraine, create extreme risk for patients with ESKD whose survival depends on the availability of reliable sources of clean water, electricity, transportation, and trained staff. History also shows that those treated by peritoneal dialysis and those with kidney transplants are not spared during conflicts.

Disaster planning remains critical as new COVID strains emerge, hostilities in Ukraine continue, and climate change strengthens storms. In evaluating the current status of emergency planning, stakeholders must recognize that normal is simply the time before the next disaster.

### The Disaster That Defined Failure: Hurricane Katrina

In August 2005, Hurricane Katrina struck the Northern Gulf Coast states. Katrina was directly responsible for 1833 fatalities and more than $100 billion in damage.^1^ The Gulf Coast's health care system was vulnerable before the storm: In 2003, patients with ESKD were disproportionately represented among the 21% of adults in Louisiana who were uninsured.^5,6^ After the hurricane's landfall, 94 dialysis facilities closed for at least one week, including 54 of 150 in Louisiana.^7^ The number of patients with ESKD receiving dialysis fell by 18%. In New Orleans, 44% of patients missed at least one and almost 17% missed three or more dialysis sessions; the adjusted odds ratio for hospitalization among the latter group was 2.16.^8^

Overall, kidney patients had poorer health outcomes because of broad lapses in the federal health care safety net, disruptions in dialysis center emergency operations, and lack of personal emergency preparation. State-based Medicaid programs maintained restrictive eligibility rules which shut out patients with ESKD.^9^ Unstable housing, lack of an easily accessible patient database, and overwhelmed landline and cellular telephone networks^9^ prevented dialysis facilities from tracking patients. Facility evacuation plans contributed to missed dialysis appointments. Many patients did not have paper copies of medical and personal information, contacts for alternate dialysis units, or a 2-week supply of medications and a nonperishable renal diet.

### A Decade and a Half of Progress

The health effects of Hurricane Katrina underscored the need for more robust disaster preparedness. Regulatory changes to Medicare and Medicaid and creation of the Administration for Strategic Preparedness and Response^10^ along with increased proactivity in dialysis facilities placed a new emphasis on the whole community approach to disaster management.

Five years after Hurricane Katrina, the Affordable Care Act expanded Medicaid eligibility to all Americans earning <133% of the federal poverty level.^11^ Had this legislation existed at the time, the overwhelming majority of Hurricane Katrina victims would have been eligible. A decade after its passage into law, 20 million Americans gained coverage.^12^ Although the Affordable Care Act has strengthened the health care safety net, its benefits have not been fully realized because most of the Gulf states affected in 2005 have not adopted the optional Medicaid expansion.^13^

The Centers for Medicare and Medicaid Services requires dialysis providers to have actionable disaster preparedness plans. Biannual audits assess four components: risk assessment and emergency planning, communication plan, policies and procedures, and training with table-top testing.^14^ Providers must demonstrate the capacity to address equipment, power, and water supply failures and ensure the continuity of patient care through coordination with other facilities. Facilities must maintain annual contact with local disaster management agencies to safeguard the needs of patients with ESKD during disasters.

Dialysis providers offer patient education before a disaster, which provided enormous benefits after Hurricane Sandy in 2012. Treatments provided before Sandy made landfall significantly decreased ED visits, hospitalizations, and 30-day mortality.^15^ These successes would not have happened without improved disaster response systems and input from state health officials who encouraged rapid activation of the Kidney Community Emergency Response Coalition^15^ which was formed in the aftermath of Hurricane Katrina, to collaboratively develop, disseminate, implement, and maintain a coordinated preparedness and response framework for the kidney community. It is tasked with raising public awareness of the specific needs of individuals with kidney disease and promoting planning for dialysis services ahead of emergencies. The Kidney Community Emergency Response Coalition supports patients' personal disaster preparedness using educational webinars, pamphlets, and social media.

### Charting a Path through the Coronavirus Pandemic

Patients with ESKD suffered disproportionate rates of COVID-19 hospitalization and fatality,^16^ especially during the early days of the pandemic. They remain at risk because of new variants, vaccine hesitancy, and questions about the effectiveness of vaccines.

### Finding Care during War

Visiting a dialysis center may not be feasible in an active war zone. The bombing of Mariupol during the war in Ukraine killed most patients with ESKD: One survivor reported that 49 of 50 patients in the dialysis center died. The World Health Organization's Surveillance System for Attacks on Health Care documented 186 attacks on health care facilities including dialysis centers, ambulances, and medical warehouses through May 3, 2022.^17^ During the Iraqi occupation of Kuwait, the mortality of patients with ESKD remaining in Kuwait was four times greater than those who evacuated.^18^

Patients who evacuate need to navigate foreign health systems. The Polish Government announced that Ukrainian refugees would be treated similarly to their own citizens, and care would be covered by the Polish National Health Fund. Unfortunately, not all countries receiving evacuees have been as generous. In Syria, government support is not available in opposition-controlled areas; patients rely on nongovernmental organizations and private donors who lack information about dialysis operations.^19^ A 2015 survey of Syrian refugees in Jordan found that 25% of patients did not receive dialysis for at least a week, mostly because of financial constraints, and 46% of patients moved at least three times to access care. During the occupation of Kuwait, patients on automated peritoneal dialysis had 95% mortality, so the remaining patients switched to manual therapies.^20^

Governments, providers, and patients must approach disaster planning to promote resilience. Learning from the pitfalls of these events, we put forth these recommendations as broad strokes to improve health outcomes for patients with ESKD and to address barriers to patient care and well-being, especially in underserved communities.

### Federal, State, and Local Governments

#### 1. Minimize the financial burden on patients

Although social safety nets have expanded since Hurricane Katrina, during the COVID-19 pandemic, millions of people suffered job loss accompanied by loss of medical coverage. The same segments of the population most affected are most likely to be exposed to and die from COVID-19. These underserved communities have disproportionate rates of chronic health conditions including ESKD.^21^ These compounding issues make life especially difficult for patients with ESKD, who might face up to $10,000 in health care costs if hospitalized with COVID-19.^22^

To reduce the effect of the pandemic, Congress passed the Families First Coronavirus Response Act,^23^ which prevents insurers from charging copayments or applying deductibles to coronavirus tests, and the CARES Act^24^ to pay for out-of-network coronavirus tests. However, nationwide, 2.4% of coronavirus tests billed to insurers leave patients responsible for significant costs.^25^

#### 2. Invest in broadband access for telehealth expansion

Although the pandemic has caused immense disruption and suffering, the crisis also provides opportunities to modernize health care delivery. During the public health emergency, regulatory waivers allowed providers to deliver and bill for services across state lines^26^ and use a variety of videoconferencing platforms to conduct virtual appointments with patients. Telehealth for patients with ESKD grants greater home-based care, less travel time, fewer trips to the clinic, increased home dialysis education, and greater patient autonomy and self-care.^27^

Telemedicine is likely to become a norm for health care delivery. However, lack of broadband access is a significant barrier for rural, underserved, and older populations. Only 43% of those aged 65 years or older, 70% of urban Americans, and 62% of rural residents have broadband access at home.^28^ Racial disparities, levels of education, and income correlate with lack of access for telehealth visits. Necessary digital equipment must also be readily available, and solutions, such as nonprofit partnerships, to redistribute refurbished devices should be pursued.

### Dialysis Providers

#### 1. Re-evaluate operational protocols

The foremost challenge of the COVID-19 pandemic is ensuring that infected patients and staff do not expose others during transportation to, and treatment at, dialysis facilities. Facilities had to adapt: changing patients' scheduled treatments, enhancing cleaning of treatment stations, minimizing the time patients spend in waiting areas, maximizing the distance between them, and changing placement of tissues and waste receptacles. Scheduling and spacing of staff breaks were also critical.^29^

#### 2. Ensure self-sufficiency for vaccine distribution

Because of their multiple comorbid conditions and inability to reduce exposure by staying at home, patients with ESKD were encouraged by their dialysis providers to receive early vaccination against COVID-19.^30^ Working collaboratively with the Centers for Disease Control and Prevention, the White House, and the American Society of Nephrology, providers distributed vaccines to all patients quickly, and uptake was high among this vulnerable population.^31^

### Patients

#### 1. Improve digital literacy

Telemedicine appointments are more convenient and encourage greater patient autonomy, but adjusting to the technology can be a significant barrier to wide adoption. Free and low-cost educational resources on technology basics are available. Patients with ESKD can engage with kidney community social media pages and attend educational workshops online.^32^ Using this crisis as an opportunity to become tech savvy is an investment in one's health and well-being that will pay off as health care becomes increasingly digital.

#### 2. Invest in mental health

Disasters wreak havoc on mental health and can make coping with everyday necessities difficult. The psychological toll is disturbingly evident years after Hurricane Katrina, with rates of anxiety, depression, post-traumatic stress disorder, addiction, domestic violence, and murder all significantly higher than the years before the storm.^33^

The COVID-19 pandemic had unprecedented geographic scope and human toll. No one is immune from the disruption of normalcy, sense of danger, social isolation, financial instability, trauma of hospitalization, and loss of hundreds of millions of lives. Patients with ESKD and their caregivers must fortify coping strategies to maintain the behaviors that prevent exposure to the virus and bolster their overall health.

One positive element of this crisis is that discussing mental health has become more mainstream and less stigmatized. Many free resources and tools are available to add to a healthy lifestyle. App-based guided meditations, communal streaming platforms, and online therapy can be incorporated into an everyday mental wellness routine. Engaging with kidney community social networks can help alleviate the particular stress and anxieties of patients with ESKD.

## Final Thoughts

Disasters like Hurricane Katrina, the coronavirus pandemic, and human conflict shock our nation's health care system and lead to dire consequences. However, they offer a chance to rebuild a more efficient, equitable, and resilient system. Preparation and communication are crucial to ensuring that patients have access to essential health care. Although major changes after Hurricane Katrina led to great improvements, preparation remained inadequate at all levels for the COVID-19 pandemic. Taking from the lessons learned from those on the frontlines battling these disasters, our recommendations can mitigate complications and ameliorate mortality in highly vulnerable patients.
